# Skin concerns and treatment seeking trends among patients visiting aesthetic clinics in Lahore, Pakistan

**DOI:** 10.12669/pjms.42.9.13943

**Published:** 2026-09

**Authors:** Muhammad Awais Arif, Shamsa Kanwal, Areesha Arif

**Affiliations:** 1 M. Awais Arif, My Skin Aesthetics and Laser Center, Lahore, Pakistan; 2 Shamsa KanwalDepartment of Computer Sciences, Muhammad Ali Jinnah University, Karachi, Pakistan; 3 Areesha Arif, My Skin Aesthetics and Laser Center, Lahore, Pakistan

**Keywords:** Aesthetics, Acne, Botulinum Toxin, Dermatology, Fillers, Melasma, Pigmentation, Scars

## Abstract

**Background & Objective:**

The increasing demand for aesthetic services, combined with the lucrative nature of the aesthetic industry, has led many healthcare providers to enter this field. This led to the rapid expansion of aesthetic clinics worldwide. However, many of these establishments face early closures. The primary objective of this study was to identify the most prevalent skin concerns among patients visiting dermatology and aesthetic clinics. In addition, the study aims to analyze patients’ treatment-seeking behavior and trends.

**Methodology:**

This retrospective observational study was conducted in two branches of an aesthetic and dermatology clinic in Lahore, Pakistan, from June 2019 to December 2023, covering 6,189 patients. Data was analyzed using SPSS version 21, focusing on identifying patients’ presenting complaints and demographic trends.

**Results:**

The study revealed that almost half of the patients (45%) visited clinics related to pigmentation concerns, while acne-related concerns accounted for 27% of patient visits. Notably, only 4.5% of patients directly sought aesthetic services, including fillers and botulinum toxin injections. Pigmentation was the most frequently reported skin concern (45.2%), followed by scars (13.8%) and acne (12.8%). The majority patients were females (69%) aged 15-34 years and primarily presented with pigmentation and hirsutism, while males (31%) primarily sought treatments for acne, acne scars, and hair loss.

**Conclusion:**

The findings of this study offer valuable insight into patient trends and treatment-seeking behavior within aesthetic practice. Unlike government or tertiary care hospital-based studies, this study uniquely reflects skin concerns in private dermatology and aesthetic clinics. We hypothesize that specialized dermatological qualifications may contribute to the success of private aesthetic practice; however, this hypothesis was not directly tested in the present study and warrants further investigation in future research.

## INTRODUCTION

Aesthetics has become a significant phenomenon that has recently transformed the beauty industry. Globally, 123 million aesthetic treatments were performed in 2023, with total procedure fees of $47 billion for these treatments.^1^ In the United States alone, in 2022, the revenue of aesthetic procedures was $11.8 billion: $8.8 billion for surgical procedures and $3.3 billion for non-surgical procedures. The top non-surgical procedure was neurotoxin, which accounted for 39% of the total revenue. A 14% surge in aesthetic procedures was noticed in 2022 compared to 2021, and there was a 58% rise in non-surgical procedures between 2018 and 2022.^2^ Aesthetic treatments such as botulinum toxin (Botox) are widely utilized in Pakistan; however, their adoption is limited due to the concerns regarding their adverse effects. A 2024 cross-sectional study reported that 63.9% of participants (n=262) demonstrated adequate knowledge of botulinum toxin injections.^3^ Acne is the most common skin condition in Pakistan, affecting 23.3% of a study group of 300 individuals, with variations by age and socioeconomic status.^4^

These statistics attract new doctors who want to establish their clinics or practice independently. The growing demand for aesthetics has led many to enter this field and open their aesthetic clinics. Aesthetics is a specialty primarily focused on the treatment of skin and underlying tissues and is typically pursued by dermatologists or plastic surgeons. The excessive use of multi-step skincare regimens by patients influenced by the social media content has also become a growing concern among dermatologists, as it is often associated with cutaneous side effects such as irritation and allergic reactions.^3^

The situation is the same in developing countries, including Pakistan. In recent years, new aesthetic clinics have proliferated in the country. This study will analyze patients’ treatment-seeking behavior and trends at the aesthetic clinics.

To figure out the treatment seeking behavior of patients at aesthetic clinics, data from the author’s own dermatology and aesthetic clinics was analyzed. The data collected was of utmost importance in understanding patients’ treatment-seeking behavior, particularly since there is little literature published on the prevalence of patients presenting to a private aesthetic clinic. The results of this study will assist new doctors in making informed decisions about the key factors to consider in the process.

## METHODOLOGY

This retrospective observational study was conducted by analyzing clinical data of patients presenting at two branches of the author’s privately operated dermatology and aesthetic clinics in Lahore, Pakistan (My Skin Aesthetics and Laser Center) during the period from 21^st^ June 2019 to 31^st^ December 2023. All new patients attending either branch for their skin concerns were included in the study. The distribution of patients was analyzed according to their self-reported presenting complaints (symptoms). All new patients visiting for the first time at the clinic were included in the study, while all retreatment patients and any duplicate record were excluded.

### Ethical approval:

Ethical approval was obtained from the research ethics committee at My Skin Aesthetics and Laser Center on March 12,2024; Ref: MS-002/24.

### Statistical analysis:

Patient data was collected from clinic records, which were then entered into SPSS version 21 for advanced analysis. The data was analyzed for the assessment of the pattern of the patient’s presenting complaints (symptoms). The assessment included variables such as age, gender, symptoms, and enrollment branch. For quantitative variables (age), mean and standard deviation were calculated, while percentage was used to report qualitative variables (gender and symptoms). Comparisons of categorical variables were made using Pearson’s chi-square test. A *p-value* of <0.05 was considered significant.

## RESULTS

During the study period, a total of 6,189 cases of dermatology were retrieved from the records of DHA and Shadman clinics. The pattern of symptoms observed in our study is shown in Table-I. Out of 6,189 cases, pigmentation was the most common skin concern, affecting 2,796 (45.2%) of patients. Acne and acne scarring accounted for the second most common concern, representing 12.8% and 13.8% respectively, followed by general skin concerns, 7.6%, and hirsutism, 4.7%. One of the key findings of this study was only 4.5% of patients visited clinics seeking aesthetic treatments, including fillers and botulinum toxin injections, with aesthetic concerns documented in 101 patients (1.6%) and fillers and botulinum toxin in 180 patients (2.9%). The distribution of presenting complaints differed significantly between the DHA and Shadman clinics across all symptom categories (p<0.05)

**Table-I:** Distribution of presenting complaints (symptoms) by Clinic Location and Gender.

Symptoms	Total (n=6189)	Clinic Location		Gender	
		DHA, n (%)	Shadman, n (%)	Female, n(%)	Male, n (%)
Acne	794(12.8)	117(8.7)	677(14.0)	537(12.5)	257(13.5)
Aesthetic concerns	101(1.6)	30(2.2)	71(1.5)	57(1.3)	44(2.3)
Fillers and Botulinum toxin	180(2.9)	48(3.6)	132(2.7)	126(2.9)	54(2.8)
Hair loss	263(4.3)	69(5.1)	194(4.0)	99(2.3)	164(8.6)
Hirsutism/Hypertrichosis	292(4.7)	46(3.4)	246(5.1)	265(6.2)	27(1.4)
Minor surgical procedures	261(4.3)	70(5.2)	191(3.9)	182(4.2)	79(4.2)
Pigmentation	2796(45.2)	651(48.4)	2145(44.3)	2176(50.7)	620(32.7)
Scars	855(13.8)	188(14.0)	667(13.8)	532(12.4)	323(17.0)
General skin concerns	472(7.6)	97(7.2)	375(7.7)	207(4.8)	265(14.0)
Miscellaneous	175(2.8)	29(2.2)	146(3.0)	111(2.6)	64(3.4)
Total	-	1345(21.7)	4844(78.3)	4292(69.3)	1897(30.7)

Gender distribution suggested a predominance of female patients which represented 69% of the total cases while male patients accounted for 31% yielding a female-to-male ratio of 2:3:1. Males were more likely to seek treatments for acne, acne scarring, and hair loss, than females while pigmentation and hirsutism were more prevalent among females.

The age and gender-based percentage distribution and their general characteristics are shown in Table-II and III.. The data showed that the mean age of patients was 31 years, with minimum and maximum ages being two and 87 years, respectively, with the majority (15-34 years) comprising the largest age group. Pigmentation was the most common skin concern among the age group 35-54 years while acne and acne scars were most common among the age groups of 15-34 years. The interest in aesthetic treatments, fillers, and botulinum toxin injections was more prominent over the age of 35, while hirsutism was a main concern among patients aged 15-34 years.

**Table-II:** Demographic characteristics of patients by year.

Characteristics	Total, n(%)	2019	2020	2021	2022	2023
***Gender (n=6189)***						
Female	4292(69.3)	679(70.1)	1038(66.8)	1286(73.2)	774(70.6)	515(63.3)
Male	1897(30.7)	289(29.9)	516(33.2)	471(26.8)	323(29.4)	298(36.7)
***Age (Years)***						
Min, Max	2, 87	11,74	7,87	2,74	14,64	3,66
Mean±SD	31±8	32±8	31±9	31±8	30±8	29±8

**Table-III:** Classification of Skin Symptom Subtypes.

Symptoms	Subtype	Total, n(%)	DHA, n(%)	Jail road, n(%)
Acne	Body Acne	19(2.4)	2(1.7)	17(2.5)
	Comedones	203(25.6)	28(23.9)	175(25.8)
	Facial acne	572(72.0)	87(74.4)	485(71.6)
Aesthetic Concerns	Anti-aging	18(17.8)	4(13.3)	14(19.7)
	Cosmetic concerns	48(47.5)	14(46.7)	34(47.9)
	Fat lysis concerns	32(31.7)	11(36.7)	21(29.6)
	Under Eye Bags	3(3.0)	1(3.3)	2(2.8)
Fillers and Botulinum Toxin	Botox	56(31.1)	14(29.2)	42(31.8)
	Botox Eyebrows Lifting	9(5.0)	1(2.1)	8(6.1)
	Botox Upper Face	36(20.0)	9(18.8)	27(20.5)
	Fillers	11(6.1)	4(8.3)	7(5.3)
	Nasolabial Fold Fillers	18(10.0)	8(16.7)	10(7.6)
	Under Eye Fillers	50(27.8)	12(25.0)	38(28.8)
Hair Loss	Hair Loss	263(100)	69(100)	194(100)
Hirsutism/ Hypertrichosis	Body Hair	6(2.1)	3(6.5)	3(1.2)
	Facial Hair	286(97.9)	43(93.5)	243(98.8)
Minor Surgical Procedures	Mole Removal	200(76.6)	57(81.4)	143(74.9)
	Skin Tag Removal	36(13.8)	8(11.4)	28(14.7)
	Wart Removal	25(9.6)	5(7.1)	20(10.5)
Pigmentation	Body Pigmentation	9(0.3)	4(0.6)	5(0.2)
	Dark Spots	650(23.2)	165(25.3)	485(22.6)
	Facial Pigmentation	777(27.8)	150(23.0)	627(29.2)
	Freckles	70(2.5)	23(3.5)	47(2.2)
	Lip Pigmentation	42(1.5)	15(2.3)	27(1.3)
	Melasma	1198(42.8)	283(43.5)	915(42.7)
	Under eye Pigmentation	50(1.8)	11(1.7)	39(1.8)
Scars	Acne Scars	795(93.0)	177(94.1)	618(92.7)
	Other Scars	60(7.0)	11(5.8)	49(7.3)
General Skin Concerns	Birthmarks	46(9.7)	10(10.3)	36(9.6)
	Facial Redness	37(7.8)	8(8.2)	29(7.7)
	Skin Diseases	343(72.7)	65(67.0)	278(74.1)
	Xerosis	46(9.7)	14(14.4)	32(8.5)
Miscellaneous	Low-Frequency Concerns	175(100)	29(100)	146(100)

The analysis showed that nearly 70% of clinic visitors were female, and their presenting complaints often differed from males. Among aesthetic concerns, most patients sought treatments for cosmetic concerns, with the majority requesting HydraFacial and tattoo removal. Among minor surgical procedures, most patients sought treatment for mole removal.

Patients seeking botulinum toxin treatments typically requested Botox, while those seeking fillers most asked for under-eye fillers and nasolabial fold fillers. Patients requesting treatment for pigmentation and acne, the majority were presented with melasma and facial acne respectively. Furthermore, patients with hirsutism mostly requested facial hair removal treatment.

In Table-IV, details the distribution of skin concerns according to age groups. Acne was the most common concern among those aged 15-34 (87.8%) and decreased significantly among the older age groups, with only 0.4% among those aged ≥55. Similarly, scars were a major skin issue among patients visiting the clinics in the age group 15-34 (85.5%). Overall acne and scars were the most reported skin concerns among the younger population aged less than 35 years.

**Table-IV:** Distribution of Skin Symptoms by Age Group.

Age (Years)	Symptoms									
	Acne n= 794	Aesthetic concerns n= 101	Fillers and Botox n= 180	Hair loss n= 263	Hirsutism/ Hypertrichosis n= 292	Minor surgical procedures n= 261	Pigmentation n= 2796	Scars n= 855	General skin concerns n= 472	Miscellaneous n= 175
≤14	5 (0.6)	2 (2.0)	1 (0.6)	4 (1.5)	0	2 (0.8)	6 (0.2)	3 (0.4)	7 (1.5)	3 (1.7)
15-34	697 (87.8)	57 (56.4)	70 (38.9)	209 (79.5)	248 (84.9)	182 (69.7)	1933 (69.1)	731 (85.5)	322 (68.2)	126 (72.0)
35-54	89 (11.2)	35 (34.7)	89 (49.4)	47 (17.9)	42 (14.4)	64 (24.5)	804 (28.8)	116 (13.6)	113 (23.9)	38 (21.7)
≥55	3 (0.4)	7 (6.9)	20 (11.1)	3 (1.1)	2 (0.7)	13 (5.0)	53 (1.9)	5 (0.6)	30 (6.4)	8 (4.6)

Aesthetic concerns were commonly sought after by individuals aged 15-34 (56.4%) while fillers and botulinum treatments were primarily asked by those aged 35-54 (49.4%). Pigmentation issues were mainly reported by the 15-34 group (69.1%). Nearly 98% of pigmentation concerns were reported by the age groups 15-34 and 35-54. Hair loss, general skin concerns, and hirsutism/hypertrichosis were notably high in younger individuals in the 15-34 group. In general, pigmentation emerged as the most reported skin concern (45.2%), followed by scars (13.8%) and acne (12.8%).

## DISCUSSION

Common dermatological conditions such as pigmentation, acne, and scars affect an estimated 100 million individuals each year, highlighting a substantial burden on aesthetic and dermatology clinics.^5^ Furthermore, Google Trends demonstrated growing patient interest in facial cosmetic procedures, providing valuable insights into patterns of patient demand.^6^ Additionally, an increasing number of individuals with skin of color are seeking aesthetic treatments, particularly for pigmentation and scarring, that collectively impact approximately 100 million people annually.^7^ The trend is particularly notable among women, with consultations for hair-related disorders rising from 1.24% in 2010 to 9.44% in 2020.^8^,^9^

This study provides valuable insight into patients’ treatment-seeking behavior and their preferences for visiting an aesthetic and a dermatology clinic. It is one of the few studies to examine patients’ treatment-seeking behavior in the private setting of an aesthetic clinic, whereas most published data focus on dermatological ailments in hospital-based public-sector settings. The study also provides insights into patients’ treatment-seeking trends, their preferences for visiting an aesthetic clinic, the most common skin concerns, and valuable considerations for a new healthcare provider planning to open an aesthetic practice.

A key finding of this study is that nearly 45% of patients visited the clinic for pigmentation issues, followed by 27% acne and scars. Only 4.5% sought aesthetic treatments like fillers and botulinum toxin injections. Among pigmentation concerns, most patients visited the clinics requesting treatment for melasma. This finding aligned with other regional studies that have linked melasma with hormonal changes, sun exposure, misuse of topical steroids, and low-quality skin whitening creams.^4^,^5^ Uneven skin tone and dark spots were subsequent pigmentary symptoms for which patients visited the clinics.

Our data further reveals that the prevalence of facial acne was quite high among acne patients, with almost 98% of total acne patients presenting with facial acne and comedones. This could also be influenced by using topical steroid-based creams 10,11 or hormonal fluctuations, as most patients belong to the 15-34 age group. This age group is more susceptible to hormonal changes known to cause acne.^12^ This study also shows that the prevalence of patients seeking aesthetic treatments is higher at the DHA branch than at the Shadman branch, while general skin concerns and acne patients mostly visited the Shadman branch.

Our analysis showed that nearly 20% of the symptoms, including pigmentation, acne, scars, and general skin concerns, accounted for almost 72% of patient visits to the clinic (4,917 out of 6,189). Several previously published clinical studies have also reported significant association between pigmentation, wound healing, scarring, and hirsuitism.^13^-^15^ These statistics are important for healthcare providers planning to open new aesthetic clinics. As previously discussed, these results further emphasize the importance of specialized qualifications for new doctors planning to open an aesthetic clinic.

There is an observed decline in patient visits throughout our study period which might be because, over time, the increased number of new aesthetic clinics heightened competition. Also, economic factors such as reduced purchasing power and rising inflation might have contributed to the reduced frequency of clinic visits. We hypothesize that certain economic factors could also influence aesthetic market. As during economic downturns, patients might prioritize treating key health issues over elective aesthetic procedures.

### Limitations:

Since it is a retrospective study, it cannot establish causal relationships. It is a dermatologist-led study, and patient trends in seeking aesthetic treatments may vary, as some patients prefer to visit exclusively specialized aesthetic treatment centers. Classification of patients according to their self-reported presenting symptoms may have introduced information bias. Patients’ treatment-seeking behavior in developing countries might be different from developed countries; therefore, to better understand these trends further research is needed. The analysis relied on routinely collected clinic records, which may have resulted in incomplete or inconsistently documented information. Finally, as the study was conducted within privately owned clinics in Lahore, the findings may not be generalizable to public healthcare settings or other regions.

## CONCLUSION

Aesthetic clinics are experiencing exponential growth worldwide, including in developing countries. This study provides key insights and trends in aesthetic procedures, treatment-seeking behaviors, and preferences in private settings. Unlike government or tertiary care hospital-based studies, this study uniquely reflects skin concerns in private dermatology and aesthetic clinics. Of the total patients, 70% were females, and their symptoms often differed from those of males. Nearly 20% of the symptoms, including pigmentation, acne, scars, and general skin concerns, accounted for almost 72% of patient visits, while only a small percentage (4.5%) asked for aesthetic treatments directly.

These findings suggest predominant dermatological concerns rather than purely aesthetic complaints among patients presenting to aesthetic clinics, along with notable gender-based differences in treatment seeking behaviors.

### Recommendations:

Though this was beyond the scope of the present study and should be examined in future research.

### Author’s Contribution:

**MAA:** Conceptualization and design of the study, data collection, drafting of the original manuscript, supervision of the research process, critical revision of the manuscript, and final approval of the version to be published.

**SK:** Data analysis, interpretation, critical revision of the manuscript for important intellectual content, and final approval of the version to be published and responsible for the accuracy of the study.

**AA:** Contribution to data interpretation, critical review and editing of the manuscript, and final approval of the version to be published.
